# Jejunal Polyps out of Place: A Case of Gastric Heterotopia of the Jejunum

**DOI:** 10.1155/2020/8822019

**Published:** 2020-08-20

**Authors:** Siri A. Urquhart, Nayantara Coelho-Prabhu

**Affiliations:** ^1^Division of Internal Medicine, Mayo Clinic, Rochester, MN, USA; ^2^Division of Gastroenterology and Hepatology, Mayo Clinic, Rochester, MN, USA

## Abstract

Heterotopia is the presence of normal physiologic tissue in an atypical location. Gastric heterotopia has been described in various locations throughout the gastrointestinal tract, including the small intestine. Gastric heterotopia of the small intestine typically is asymptomatic but may present in several ways with symptoms of obstruction, bleeding, perforation, intussusception, or pain. However, gastric heterotopia is rare beyond the duodenum except for its frequent association with Meckel's diverticulum. This entity should be considered in the differential diagnosis of polypoid lesions presenting with symptoms of bleeding or obstruction especially in younger patients. We present a case of gastric heterotopia of the jejunum in a patient with a prior history of Meckel's diverticulectomy after he presented with obstructive symptoms. His symptoms improved following resection of two jejunal polyps via antegrade double-balloon assisted enteroscopy with fluoroscopy. On histopathlogical examination, findings were consistent with gastric heterotopia. This case highlights the importance of considering gastric heterotopia in the differential diagnosis of polypoid lesions located beyond the ligament of Treitz in younger patients presenting with obstructive symptoms.

## 1. Introduction

Heterotopia is the presence of mature physiologic tissue in an atypical location. Gastric heterotopia has been described in several locations throughout the gastrointestinal tract such as the esophagus, duodenum, gallbladder, Meckel's diverticulum, and other areas within the small bowel and rectum. Gastric heterotopia of the small intestine can be asymptomatic or present in various ways with symptoms of obstruction, ulceration or bleeding, perforation, intussusception, or pain [1]. Gastric heterotopia beyond the ligament of Treitz is rare but should be considered in the differential diagnosis of polypoid lesions in young patients presenting with gastrointestinal bleeding or symptoms of obstruction [2].

## 2. Case Presentation

A 33-year-old gentleman presented with lower abdominal pain, occasional nausea, emesis, and inability to pass stool or flatus. He did not have any fever or chills. His past medical history was notable for Meckel's diverticulectomy secondary to gastrointestinal bleeding which ultimately required ileal resection and stapled anastomosis ten years previously. The pathology following operative intervention did not demonstrate any ectopic gastric mucosa. He did not have any additional pertinent past medical, family, or social history.

**Figure 1:**
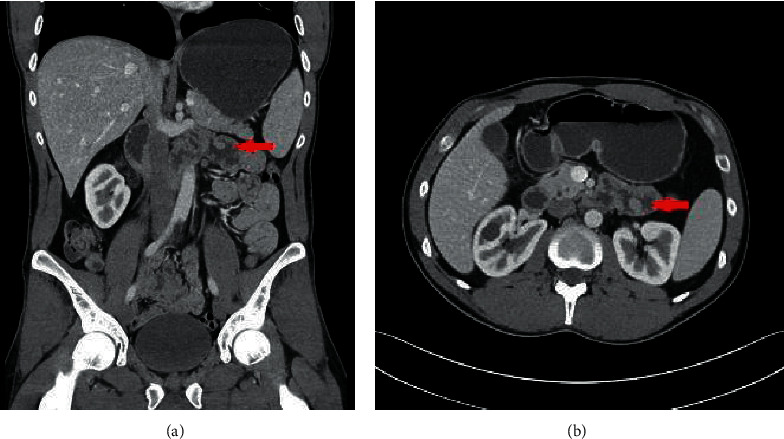
Coronal (a) and axial (b) computed tomography enterography of the abdomen and pelvis with contrast demonstrating a 0.8 cm jejunal polyp in the proximal jejunum just past the ligament of Treitz.

**Figure 2:**
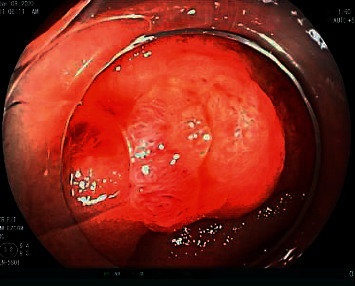
Antegrade double-balloon assisted enteroscopy with fluoroscopy demonstrating a semisessile polyp without bleeding at the ligament of Treitz.

**Figure 3:**
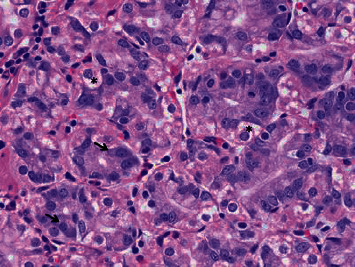
Histopathology with nodular areas of gastric fundic heterotopia without dysplasia. Oxyntic glands with chief cells (arrows) and parietal cells (asterisks) are shown at high magnification.

His vital signs were within normal limits. Physical examination was notable for a minimally distended abdomen with tympany on percussion. Laboratory investigations were unremarkable. Coronal and axial computed tomography enterography of the abdomen and pelvis with contrast (Figure 1) demonstrated a 0.8 cm jejunal polyp in the proximal jejunum just past the ligament of Treitz. He subsequently underwent an antegrade double-balloon assisted enteroscopy with fluoroscopy which revealed a 1.5 cm semisessile polyp without bleeding at the ligament of Treitz in addition to a 0.7 cm sessile polyp in the proximal jejunum (Figure 2) which were then resected. Histopathology demonstrated nodular areas of gastric fundic heterotopia without dysplasia. Oxyntic glands with chief cells (arrows) and parietal cells (asterisks) are shown at high magnification (Figure 3). A diagnosis of gastric heterotopia of the jejunum was made. Following endoscopic resection, the patient was advised to avoid aspirin or nonsteroidal anti-inflammatory medications and to monitor hemoglobin annually for anemia.

## 3. Discussion

Heterotopia is defined as the presence of normal physiologic tissue in an anatomic location where it is not normally found. Gastric heterotopia is not an uncommon lesion and can be found in several areas throughout the gastrointestinal tract. However, gastric heterotopia is rare beyond the duodenum except for its frequent association with Meckel's diverticulum [3]. Gastric heterotopia of the small intestine may be asymptomatic or present with symptoms of intestinal obstruction, ulceration or bleeding, perforation, intussusception, or pain [1]. Gross appearance is characteristically a mucosal nodularity or polypoid lesion [2, 4]. Peptic ulceration in the area of heterotopia followed by inflammation and fibrosis may result in the formation of a stricture. Polyps can cause intussusception. On microscopic examination, the surface is lined by gastric foveolar epithelium with gastric glands, typically with fundic type mucosa [2].

It is important to differentiate gastric heterotopia from gastric metaplasia. Gastric metaplasia is an acquired lesion and is usually seen in association with chronic inflammatory conditions such as inflammatory bowel disease. Unlike heterotopia which is a macroscopic lesion seen on radiographic imaging or endoscopy, ultimately requiring confirmation by biopsy, metaplasia is a microscopic lesion [2].

The typical treatment for gastric heterotopia includes endoscopic or surgical resection to prevent complications. This case is unique in that the patient had two foci of gastric tissue outside of the stomach with a prior history of Meckel's diverticulectomy and prior pathology at the time of small bowel resection without any evidence of ectopic gastric mucosa.

It is important to recognize the variety of presentations associated with gastric heterotopia. A timely diagnosis of an enlarged polyp can prevent complications such as obstruction, intussusception, bleeding, or perforation. The combination of clinical presentation, radiologic imaging, and endoscopic and pathologic evaluation is helpful in making the diagnosis [5].

In most reported cases of gastric heterotopia involving the jejunum (Table 1), patients had a median age of 21.5 years with the youngest patient being one-year-old [6]. Presenting symptoms consisted of gastrointestinal bleeding [7–9] or obstructive symptoms as a result of a polypoid mass [1, 5, 10–17] and stricture [2, 3, 6]. One case report described a 16-year-old patient who developed perforation and ulceration involving the jejunum in the setting of gastric heterotopia [18]. One case report described a 21-year-old patient with a 15 cm intraluminal polypoid mass involving the jejunum [15], while two case reports described multiple strictures associated with jejunal gastric heterotopia [2, 6]. Gastric heterotopia was not suspected clinically in any of these cases and was diagnosed on histopathological examination.

Gastric heterotopia can present in various ways including masslike lesions with symptoms of obstruction, pain, or bleeding, or may even remain asymptomatic. Gastric heterotopia beyond the ligament of Treitz is a rare entity. This case highlights the importance of considering gastric heterotopia in the differential diagnosis of polypoid lesions located beyond the ligament of Treitz in younger patients presenting with obstructive symptoms [2].

## Figures and Tables

**Table 1:** Summary of previously reported cases of gastric heterotopia of the jejunum.

Author	Age	Sex	Presenting features and diagnosis	Duration	Gross appearance	Size	Treatment
Ahn, et al. [10]	5	F	Vomiting, abdominal pain; diagnosed with intussusception of proximal jejunal loops	2 weeks	Polypoid lesions with erosions on the proximal jejunum	Variable-sized with largest nearly obstructing jejunal lumen	Endoscopic resection
Ali, et al. [7]	23	M	Melena, nausea, and vomiting	Unknown	Jejunal diverticulum with white mucosal plaques 50 cm from pylorus	Unknown	Midline laparotomy and endoscopic wedge resection
Al-Jaadan, et al. [6]	1	F	Episodic abdominal distension, vomiting, diarrhea, and failure to thrive	3 years	Dilated segment of proximal jejunum followed by 8 cm narrowed segment with thickened walls	38 cm dilated segment of proximal jejunum	Laparotomy with en-bloc small bowel resection
Bhattacharya, et al. [1]	52	F	Intermittent cramping, abdominal pain, and vomiting	Unknown	Mucosal, broad-based, polypoid mass	4 × 2.7 × 0.4 cm	Exploratory laparotomy with partial resection of jejunum
Chinnery, et al. [3]	17	F	Postprandial vomiting and weight loss	3 months	Jejunal stricture 15 cm distal to ligament of Treitz	2 cm in length	Surgical excision
Isbister, et al. [18]	16	F	Abdominal pain and vomiting	1 week	Perforation and ulcer involving the jejunum, 25 cm from duodenojejunal flexure	2.5 cm in diameter	Laparotomy with surgical resection
Jimenez, et al. [8]	4	M	Abdominal pain and melena	3 days	Large polypoid mass occupying 50% of the lumen	9 × 4 cm	Laparotomy with en-bloc small bowel resection
Khan, et al. [11]	36	F	Abdominal pain and vomiting	Unknown	Polypoid lesion	2 cm	Laparoscopic resection
Kimpton, et al. [12]	7	F	Episodic abdominal pain, nausea, and vomiting, and intussusception	4 years	Tumor mass filling the entire lumen of the jejunum	3 cm in diameter	Surgical resection
Lee, et al. [13]	25	M	Postprandial abdominal pain and vomiting	3 weeks	Elongated, serpiginous mucosal tumors	8 × 3 cm and 6 × 3 cm	Surgical resection
Leng, et al. [9]	9	F	Gastrointestinal bleeding with melena and mild anemia	15 months	Polypoid mass	4 cm	Laparoscopic resection
Mandrekar, et al. [14]	22	F	Intestinal obstruction	Unknown	Polypoidal mass	8 × 6 × 2 cm	Emergency laparotomy and resection
Martinez, et al. [15]	21	F	Intermittent abdominal pain, nausea, and vomiting	1 year	Large intraluminal tumor of the jejunum	15 cm	Abdominal laparotomy with resection
Nasir, et al. [5]	31	M	Postprandial abdominal pain and hematochezia	10 years	Polypoid mass at duodenojejunal junction	6 × 2.5 cm	Endoscopic resection
Nwanze, et al. [16]	24	F	Abdominal pain, nausea and vomiting, and intussusception	12 hours	Protruding polypoid mass of the jejunum	3.4 × 2.7 × 2.4 cm	Emergent surgical resection
Omotosho, et al. [17]	17	F	Refluxlike symptoms, abdominal pain, vomiting, and intussusception	6 months	Bilobed intraluminal jejunal polyp	Unknown	Surgical resection
Vani, et al. [2]	24	M	Abdominal pain and peritonitis	Unknown	Jejunal strictures and perforation	40 cm of jejunum with multiple strictures and serosal exudate	Emergent exploratory laparotomy and resection

## Data Availability

No data were used to support this study.

## References

[1] Bhattacharya B., Jakate S., Saclarides T. J., Keshavarzian A. (2003). Gastric heterotopia presenting as a mass in jejunum. *Archives of Pathology & Laboratory Medicine*.

[2] Vani M., Nambiar A., Geetha K., Kundil B. (2017). Jejunal gastric heterotopia causing multiple strictures and perforation peritonitis: a case report with review of literature. *Journal of Clinical and Diagnostic Research*.

[3] Chinnery G. E., Bernon M. M., Banderker M. A., Roberts R., Krige J. E. J. (2013). Gastric heterotopia causing jejunal ulceration and obstruction. *South African Journal of Surgery*.

[4] Cai J., Yu H. (2017). Giant polypoid gastric heterotopia in the small intestine in a boy: a case report and literature review. *Medicine*.

[5] Nasir A., Amateau S. K., Khan S., Simpson R. W., Snover D. C., Amin K. (2018). The many faces of intestinal tract gastric heterotopia; a series of four cases highlighting clinical and pathological heterogeneity. *Human Pathology*.

[6] Al-Jadaan S., Oda O. (2014). A rare clinical presentation of heterotopic gastric mucosa of the jejunum: a case report and review of the literature. *Journal of Pediatric Surgery Case Reports*.

[7] Ali S. M., Ahmed A. A., Saaid L. (2019). Heterotopic gastric mucosa presenting as lower gastrointestinal bleeding: an unusual case report. *Case Reports in Surgery*.

[8] Jimenez J. C., Emil S., Steinmetz B., Romansky S., Weller M. (2005). Recurrent gastrointestinal tract bleeding secondary to jejunal gastric heterotopia. *Journal of Pediatric Surgery*.

[9] Leng S., Ghionzoli M., Caporalini C., Buccoliero A. M. (2016). Long-term intestinal bleeding in a child: a rare case of heterotopic gastric mucosa in the jejunum. *BMJ Case Reports*.

[10] Ahn K. R., Koo J. S., Kim H. I. (2017). Endoscopic treatment of Jejunal heterotopic gastric mucosa that caused recurrent intussusception. *Clinical Endoscopy*.

[11] Khan M. J., Mullerat P., Desai A. (2009). A polypoid gastric heterotopia of jejunum diagnosed by capsule endoscopy. *Journal of the College of Physicians and Surgeons*.

[12] Kimpton A. R., Crane A. R. (1938). Heterotopic gastric mucosa. *New England Journal of Medicine*.

[13] Lee S. M., Mosenthal W. T., Weismann R. E. (1970). Tumorous heterotopic gastric mucosa in the small intestine. *Archives of Surgery*.

[14] Mandrekar S. R., Sangeeta A., Sanjyot N., Pinto R. G. (2016). Giant polypoid gastric heterotopia of jejunum. *Medical Journal of Dr. D. Y. Patil University*.

[15] Martínez A., Decanini-Terán O., Soria-Céspedes D. (2013). Polypoid and hyperplastic heterotopic gastric mucosa in the jejunum as a cause of recurrent subocclusive episodes. *Annals of Gastroenterology*.

[16] Nwanze J., Collins V., Crawford B., Nakanishi Y. (2018). A case of small bowel intussusception in an adult caused by heterotopic gastric mucosa in the Jejunum: a case report and review of the literature. *American Journal of Clinical Pathology*.

[17] Omotosho P. A., Varnholt H., Tirabassi M. V., Prasad R., Moriarty K. P. (2007). Giant polypoid gastric heterotopia of the jejunum presenting with intermittent intussusception. *Journal of Laparoendoscopic & Advanced Surgical Techniques*.

[18] Isbister W. H., Weedon D. (1976). Perforated jejunal ulcer and heterotopic gastric mucosa. *British Journal of Surgery*.

